# Xp11.2 Translocation Renal Cell Carcinoma With *TFE3* Rearrangement: Distinct Morphological Features and Prognosis With Different Fusion Partners

**DOI:** 10.3389/fonc.2021.784993

**Published:** 2021-11-30

**Authors:** Yan Ge, Xingtao Lin, Qingling Zhang, Danyi Lin, Luqiao Luo, Huiling Wang, Zhi Li

**Affiliations:** ^1^ Department of Pathology, Guangdong Provincial People’s Hospital/Guangdong Academy of Medical Sciences, Guangzhou, China; ^2^ Department of General Surgery, Guangdong Provincial People’s Hospital/Guangdong Academy of Medical Sciences, Guangzhou, China

**Keywords:** TFE3, VCP, renal cell carcinoma, Xp11.2 translocation, rearrangement

## Abstract

**Background:**

Renal cell carcinoma (RCC) associated with Xp11.2 translocation/*TFE3* gene fusion is a rare and new subtype of RCC and was classified by the WHO in 2004. Since then, multiple 5′ fusion partners for *TFE3* have been reported; however, the impact of individual fusion variant on specific clinicopathologic features of Xp11.2 RCCs has not been well defined.

**Methods:**

Four Xp11.2 translocation RCCs were identified by morphological, immunostaining, and fluorescence *in situ* hybridization (FISH) assays from 200 patients who attended Guangdong General Hospital between January 2017 and January 2020. All these four cases were further analyzed by RNA sequencing to explore their *TFE3* gene fusion partners. The clinicopathologic features, including clinical manifestations, pathological findings, treatment strategies, clinical outcomes, and follow-up information on Xp11.2 translocation RCCs, were recorded and evaluated.

**Results:**

These four cases affected one male and three females. The median age was 13 years at the time of diagnosis (range = 4–20 years). All the examined tumors were unilateral and unifocal. The largest diameter of these tumors ranged from 2.0 to 10.0 cm, and the average was 5.55 cm. Regional lymph node or distant metastasis developed in two patients. Three cases demonstrated known fusions: *ASPCR1*–*TFE3* (two cases) and *PRCC*–*TFE3* (one case). However, one case showed an unreported *VCP*–*TFE3* fusion gene in Xp11.2 translocation RCCs. Immunohistochemistry results revealed tumor cells diffusely positive for *TFE3*, but have no consistency in other markers. Moreover, there were different clinical prognoses among the different variant *TFE3* rearrangements; RCC patients with *VCP*–*TFE3* translocation had worse prognosis compared to those with other fusion types. Follow-up were available for all the patients and ranged from 3 to 36 months. Three patients were without evidence of disease progression, while that with *VCP*–*TFE3* fusion died of the disease 3 months after the diagnosis.

**Conclusion:**

In conclusion, our data expand the list of *TFE3* gene fusion partners and the clinicopathologic features of Xp11.2 RCCs with specific *TFE3* gene fusions. We identified a novel *VCP*–*TFE3* fusion in Xp11.2 translocation RCCs for the first time, which has unique morphology and worse prognosis than those with other variant *TFE3* rearrangements. Integration of morphological, immunohistochemical, and molecular methods is often necessary for the precise diagnosis and optimal clinical management of malignant tumors.

## Introduction

Xp11.2 translocation carcinoma was first recognized as a subtype of renal cell carcinoma (RCC) in the 2004 World Health Organization classification of renal tumors (1), followed by being renamed as MiT (microphthalmia-associated transcription factor) family translocation renal cell carcinoma (tRCC) in the 2016 edition (2). Although this neoplasm is rare (1-4%) since the incidence is estimated to all renal tumors, it is frequently observed in children and adolescents and was reported to account for 20%–75% of pediatric renal neoplasms (3). The prognosis of Xp11.2 RCC is still unclear because of the low appearance of series including a great number of patients and the short follow-up period (4).

*TFE3*, a transcription factor specifically recognizing E-box sequences, is a major regulator of both Golgi and lysosomal homeostasis. Multiple 5′-fusion partners for *TFE3* have been reported. The most common gene fusion partners are *ASPL* (alias *ASPCR1*) and *PRCC*; other fusion partners include *CLTC*, *DVL2*, *LUC7L3*, *KHTFESRP*, *PARP14*, *NonO*, *SFPQ* (alias *PSF*), *MED15*, *RBM10*, *NEAT1*, *KAT6A* (5), *MATR3*, *FUBP1* (6), *SETD1B* (7), and *EWSR1* (8). The result of translocation involving the *TFE3* gene is the overexpression of the TFE3 protein. As a result of these translocations, the expression of the TFE3 fusion protein increases in the nuclei of tumor cells. Some authors have suggested that specific translocation has an influence on histological appearance (9). In this study, we used fluorescence *in situ* hybridization (FISH) to confirm four Xp11.2 tRCCs among 200 RCCs and used targeted RNA sequencing to identify the fusion genes in these four cases of Xp11.2 tRCCs. In recent years, the identification of chromosomal translocations and fusion genes has substantially contributed to diagnostic precision, enabling better understanding of the genetic mechanisms underlying carcinogenesis, thus leading to better risk stratification and the development of novel therapeutics.

## Materials and Methods

A retrospective study was performed among 200 cases diagnosed as RCC. These cases were collected from the Department of Pathology, Guangdong Provincial People’s Hospital/Guangdong Academy of Medical Sciences between January 2017 and January 2020. The clinicopathologic features, such as clinical manifestations, pathological findings, treatment strategies, clinical outcomes, and follow-up information on Xp11.2 tRCCs, were recorded and evaluated. All patients have signed an informed written consent to have their medical record data used in research.

### Immunohistochemistry

Immunohistochemistry was performed on 4-μm sections from formalin-fixed, paraffin-embedded (FFPE) tissue. The following antibodies were used, as previously described (10): HMB45 (HMB45, 1:400; Gene Tech, Shanghai, China), Melan A (A103, 1:4,000; Gene Tech, Shanghai, China), CK7 (OV-TL12/30, 1:3,200; Gene Tech, Shanghai, China), S100 (2A10, 1:400; IBL, Takasaki, Japan), cytokeratins AE1/3 (AE1/AE3, 1:400; Gene Tech, Shanghai, China), AMACR (13H4, 1:300; Gene Tech, Shanghai, China), Ki-67 (MIB-1, 1:30; BioGenex, Fremont, CA, USA), PAX8 (ZR-1, 1:800; Abcam, Cambridge, UK), CD10 (56C6, 1:200; Gene Tech, Shanghai, China), and TFE3 (MRQ-37, 1:1,000; MXB Biotechnologies, Fuzhou, China). The TFE3 antibody recognizes the C-terminal portion of the TFE3 protein. Tumor cells that were diffusely positive in nuclei were considered as strong positive. FISH detection would be recommended.

### *TFE3* Break-Apart FISH Analysis

FISH was performed on 4-μm tissue sections with two colored split-apart probes for *TFE3* (Z-2109-50; ZytoVision, Bremerhaven, Germany). The orange fluorochrome direct labeled probe hybridizes distal to the *TFE3* gene; the green fluorochrome direct labeled probe hybridizes proximal to that gene. Briefly, the tumor area on the slides was marked with a diamond-tipped pen. The slides were deparaffinized in xylene, rehydrated, treated in 750 U/ml pepsin digest solution (Sigma-Aldrich, Natick, MA, USA) for 10 min, and incubated in 10% buffered formalin for 10 min. The slides and probes were separately denatured, and hybridization was performed at 37°C overnight. Post-hybridization wash was done in 0.4× SSC/0.3% NP-40 at 73°C for 3 min, and then the slides were counterstained with DAPI. A positive score was interpreted when at least 20% of the nuclei show a split-apart signal. Nuclei with incomplete sets of signals were omitted from the scoring.

### RNA Sequencing

Four cases that demonstrated positive results for the *TFE3* break-apart FISH assay were analyzed by RNA sequencing. Total RNA from FFPE samples was extracted after xylene deparaffinization using the RNeasy FFPE Kit (Qiagen, Hilden, Germany). Ribosomal RNA was depleted using RNase H, followed by library preparation using the KAPA Stranded RNA-seq Kit with RiboErase (HMR) (KAPA Biosystems, Wilmington, MA, USA). The library concentration was calculated using a KAPA Library Quantification Kit (KAPA Biosystems), and the library quality was assessed using the Agilent High Sensitivity DNA Kit and Bioanalyzer 2100 (Agilent Technologies, Santa Clara, CA, USA), followed by sequencing on the Illumina HiSeq next-generation sequencing (NGS) platform (Illumina, San Diego, CA, USA). Three tools were used for the detection of any potential *TFE3* fusion of the RNA sequencing data. Fusion-Catcher (version 0.994e) was used with parameters (otherwise, the default parameter was used) that allowed the Bowtie aligner to perform both transcriptome and genome mapping, and the BLAT aligner was then used to further map unmapped reads and count fusion supporting evidence. The other two tools, Factera and Socrates (https://github.com/jibsch/Socrates), were both executed suing default parameters. Specifically, Socrates takes the modified BAM file, which converts the hard clip in the original BAM file into a soft clip to improve the fusion detection performance. The combined fusion results from all tools were manually reviewed on the Integrative Genomics Viewer for confirmation.

## Results

### Clinicopathologic Features

We reviewed 200 nephrectomy cases and identified four Xp11.2 tRCCs. The clinicopathological findings are summarized in **Table 1**. These four cases affected one male and three females. The median age was 13 years at the time of diagnosis (range = 4–20 years). Three tumors occurred in children. The last one occurred in an adult. All the examined tumors were unilateral and unifocal. Regional lymph node or distant metastasis developed in two patients. Follow-up was available for all patients and ranged from 3 to 36 months. Three patients were without evidence of disease progression, while one died of the disease 3 months after the diagnosis. Two patients presented with pT1 stage, two with pT2, and one with pM1.

**Table 1:** Clinicopathological characteristics of four cases of Xp11.2 renal cell carcinoma.

	Age	Sex	Side	Diameter (cm)	pT stage	Follow-up (months)	*TFE3* fusion type
1	9	Male	Right	3.0	T1aN1M0	36	*ASPL*
2	20	Female	Left	10.0	T2N0M0	36	*PRCC*
3	4	Female	Left	2.7	T2N0M0	30	*ASPL*
4	17	Female	Left	6.5	T3N?M1	3	*VCP*

**Figure 1:**
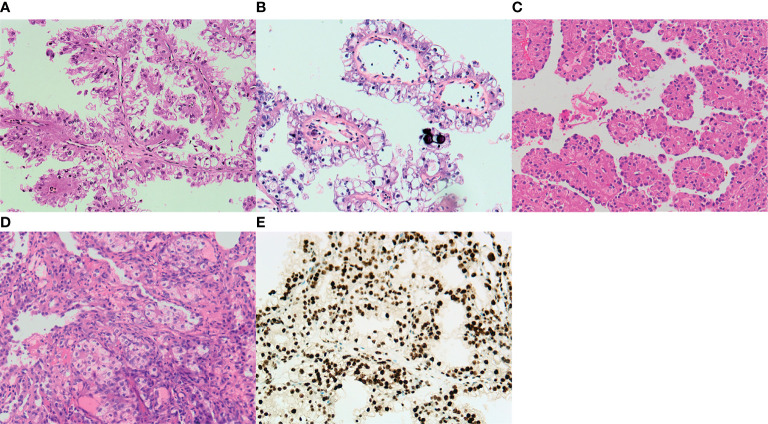
Representative images of the typical morphological features of Xp11.2 translocation renal cell carcinoma (RCC). **(A)** *ASPL–TFE3* fusion type shows a predominantly papillary morphology lined by pseudostratified clear to oncocytic cells. **(B)** Psammoma bodies are also present in *ASPL–TFE3* Xp11.2 translocation RCC. **(C)** An alveolar pattern or organoid pattern populated by eosinophilic cells is noticed in the *PRCC–TFE3* fusion type. **(D)** Xp11.2 translocation RCC with a novel fusion partner. *VCP* demonstrates a solid nested/alveolar pattern with predominant eosinophilic cells to focal clear cytoplasm. **(E)** Diagnostic TFE3 reaction strongly labeled the nuclei in the *VCP–TFE3* fusion type. All images have a magnification factor of ×200.

The largest diameter of the tumors ranged from 3.0 to 10.0 cm, with an average of 5.55 cm. Macroscopically, the cut surface was usually solid and cystic with a sulfur yellow color, the same as that seen in clear cell RCC. Foci of necrosis, hemorrhage, and peritumor calcification were occasionally noted as well. In our cohort, no rhabdoid morphology was observed. Three cases demonstrated known fusions: *ASPL*–*TFE3* (two cases) and *PRCC*–*TFE3* (one case). One case showed an unreported fusion type: *VCP*–*TFE3* in Xp11.2 tRCC. The predominant architectural appearance of the *PRCC*–*TFE3* case was solid with a predominantly eosinophilic cytoplasm. The cell size was relatively consistent, small to medium size. No psammoma body was noticed. On the other hand, the histological appearance of the *ASPL*–*TFE3* group was branching papillary composed of clear/eosinophilic cells with voluminous cytoplasm. The cell size was relatively larger than that of other fusion types. The psammoma body was easily seen. However, one case showed the *VCP*–*TFE3* fusion gene. This tumor demonstrated a solid nested/alveolar architecture featuring epithelioid cells with a predominantly eosinophilic to a focal clear cytoplasm. The morphology is very similar to that of clear cell RCC. No psammoma body was noticed. All these four cases did not have a melanin pigment. Representative pictures are displayed in **Figure 1**.

### IHC Findings, FISH Validation, and Fusion Transcripts by RNA Sequencing

The results of immunohistochemistry are summarized in **Table 2**. The four examined cases were all negative, with cytokeratin antibodies (CK7 and AE1/AE3), melanocytic markers (S-100, Melan A, and HMB45), and strong expressions of CD10, AMACR, and PAX8. The expressions of vimentin and AMACR are rare and focal. The diagnostic TFE3 reaction strongly labeled the nuclei in all cases (**Figure 1E**). FISH analysis with the *TFE3* (Xp11) break-apart probe was positive for a *TFE3* translocation.

**Table 2:** Immunohistochemistry, fluorescence *in situ* hybridization (FISH), and fusion type profiles of four cases of Xp11.2 renal cell carcinoma.

	CA-9	CK7	AE1/3	AMACR	CD10	PAX-8	S-100	Melan A	HMB45	Ki67	TFE3 IHC	TFE3 FISH	*TFE3* fusion type
1	N	N	N	F	D	F	N	N	N	3%	D	+	*ASPL*
2	N	N	N	F	D	D	N	N	N	2%	D	+	*PRCC*
3	N	F	F	F	D	D	N	N	N	10%	D	+	*ASPL*
4	N	N	N	F	D	D	N	N	N	12%	D	+	*VCP*

N, negative; F, focally positive; D, diffusely positive; IHC, immunohistochemistry.

**Figure 2:**
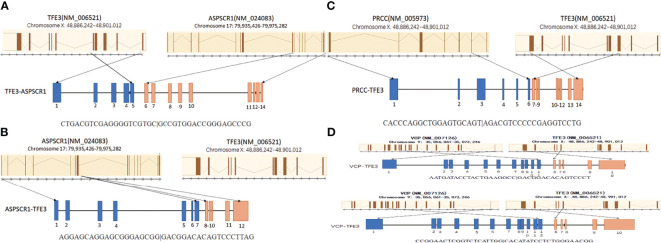
Schematic diagrams of the *TFE3* fusion transcripts with their partner genes in our cohort. **(A)** The *ASPL–TFE3* rearrangement between genes *ASPL* exon 8 and *TFE3* exon 5. **(B)** The *ASPL–TFE3* rearrangement between genes *ASPL* exon 7 and *TFE3* exon 6. **(C)** The *PRCC–TFE3* rearrangement between genes *PRCC* exon 6 and *TFE3* exon 3. **(D)** There are two *VCP–TFE3* fusion types: one between *VCP* exon 11 and *TFE3* exon 6 and the other between *VCP* exon 12 and *TFE3* exon 6.

All four neoplasms with known *TFE3* immunohistochemistry and the *TFE3* split FISH assay were analyzed by RNA sequencing for fusion partners. Gene fusions were successfully detected in four cases, of which three cases (75%) showed relatively common gene fusions, including *ASPSCR1/ASPL*–*TFE3* gene fusions (two cases) due to t(X;1)(p11.2;q21) and a *PRCC*–*TFE3* gene fusion (one case) due to t(X;17)(p11.2;q25.3). Furthermore, we found a new *VCP*–*TFE3* gene fusion due to t(x;9)(p11.23;p13.3) in our cohort (**Figure 2**).

## Discussion

Xp11.2 tRCCs have two predisposing stages: pediatric population with a mean age of 17 years (11) and the adult population with a mean age of 37 years (4). We reviewed 200 nephrectomy cases and identified four Xp11.2 tRCCs. In our study, three tumors affected children, and the oldest patient examined was 20 years old. The clinicopathological features of this cohort could probably represent the biological behavior of this rare cancer in young patients. In our cohort, the frequency of tRCC was the same as that in literature data (2.0% *vs* 1%–4%) (12). Three patients were symptomatic, and tumor-related pain was the most common symptom. Unlike other patients, the first symptom of the patient with *VCP*–*TFE3* fusion was the presence of several bone metastases. She suffered from right hip pain for 1 month, and pain was aggravated with pathological fracture for 1 day. No patient had prior history of malignancy in our study. Clinically, Xp11.2 RCCs most commonly present as a sizeable mass in the kidney. The mean size of the tumors in this study was 5.55 cm, which was smaller than that in an earlier reported series (13). An invasion of the vein and the sinus was noticed in one of the patients, the same as in the literature (14). Metastatic spread to the regional lymph nodes or distant organs was observed in two of the cases (the patient with *VCP*–*TFE3* and one of the patients with *ASPL*–*TFE3* fusion). The patient with *VCP*–*TFE3* fusion was first diagnosed at the pM1 stage.

Immunohistochemical staining is routinely used for the diagnosis of Xp11.2 tRCCs since the morphological features of these tumors commonly overlap those of other RCCs (15). TFE3 immunohistochemical staining is helpful in labeling the TFE3 protein, which is generally undetectable in normal tissues by immunohistochemistry. Moderate to strong nuclear TFE3 expression should be considered as genuinely positive, and FISH detection or RNA sequencing could be recommended. The renal tubular transcription factor PAX8 and other renal markers, such as CD10 and renal cell carcinoma marker (RCC-Mat), are consistently positive. The expressions of melanocytic markers (S100, Melan-A, and HMB-45) are frequent in *TFEB* tRCC, nevertheless are rare in Xp11.2 tRCCs (16). Unlike other adult RCCs, Xp11.2 RCC tends to underexpress epithelial markers (CK7 and AE1/AE3). A minimally positive carbonic anhydrase 9 (CA-9) signal differentiates Xp11.2 RCCs from clear cell RCCs, which consistently show diffuse CA-9 positivity (17).

Although the predominant growth patterns are papillary, tubular, nested, and mixed, the predominant histological characteristic of *TFE3*/Xp11.2 RCC is papillary architecture with clear cells and psammoma bodies. Contrasting conventional RCCs, Xp11.2 translocation-associated tumors are defined not only on a morphological but also on a genetic basis. Tumors with different specific gene fusions may have consistent, unique clinical manifestations and morphological features (18). The most common gene fusions in Xp11.2 tRCCs are the *TFE3* gene on Xp11.2 with *PRCC* at 1q21 and *TFE3* with *ASPL* at 17q25, which arise from the translocations t(X;1)(p11.2;q21) (19) and t(X;17)(p11.2;q25.3) (20). The most common subtype is *ASPL*–*TFE3* tRCC. Their morphology is mostly associated with the “classic” one: papillary/pseudopapillary architecture, large epithelial cell with voluminous cytoplasm, and psammoma bodies (21, 22). On the other hand, *PRCC*–*TFE3* tRCC is the first documented case of Xp11.2 tRCC (23, 24). The typical histological pattern is compact architecture, less voluminous cytoplasm or clearing cytoplasm, and fewer psammoma bodies. Xp11.2 tRCC with *NONO*–*TFE3* and *SFPQ*–*TFE3* are less common subtypes that show a combination of nested to papillary architecture with a secretory endometrioid appearance and subnuclear vacuoles, like clear cell papillary RCC. Psammomatous calcifications are easily noticed in these tumors (15). So far, there are only 10 reported cases of *RBM10*–*TFE3* RCCs. They have sheet, solid, papillary, and trabecular architecture, with clear to eosinophilic cytoplasm, which mimic the typical morphology of *TFEB*/t(6:11) RCC (25). Two rare cases of Xp11.2 tRCC have been reported since 2019: *MED15*–*TFE3* tRCC (26) and *NEAT1*–*TFE3* tRCC (27). Most parts of *MED15*–*TFE3* tRCCs are solid and cystic structures, but they also have a solid and small nested pattern. The cytoplasm is voluminous eosinophilic. *NEAT1*–*TFE3* tRCCs have abundant psammoma bodies and a predominantly alveolar/nested pattern. The tumors demonstrate large epithelioid and small lymphocyte-like cells. The rest of the Xp11.2 tRCCs [*CLTC*–*TFE3* (28), *DVL2*–*TFE3* (15), *LUC7L3*–*TFE3, KHSRP*–*TFE3*, *KHDRBS2*–*TFE3 (* 29), *PARP14*–*TFE3* (30), *KAT6A*–*TFE3* 27), *GRIPAP1*–*TFE3* (31)] have no distinctive histological patterns, probably because some fusion types were detected in next-generation sequencing.

However, an unreported *VCP*–*TFE3* fusion transcript due to t(x;9) (p11.23;p13.3) was found in a 17-year-old female in our study. The *VCP*–*TFE3* fusion was found between *VCP* exon 11 and *TFE3* exon 6 and between *VCP* exon 12 and *TFE3* exon 6. One article reported *VCP*–*TFE3* fusion found in a perivascular epithelioid cell tumor (PEComa) arising in the pancreas of a 69-year-old male (32). This case showed *VCP*–*TFE3* fusion between *VCP* exon 11 and *TFE3* exon 6. Our case showed a pure epithelioid morphology with a predominantly nested/alveolar (about 5% papillary/pseudopapillary) growth pattern. The tumor cells were large and polygonal with eosinophilic to clear cytoplasm. The nuclei varied from small to exceptionally large focally and demonstrated prominent nucleoli and intranuclear inclusions. No psammoma bodies or melanin pigments were noticed. Overall, the morphology of *VCP*–*TFE3* tRCC is similar to that of clear cell RCC, which is easy to be misdiagnosed. Integration of morphological, immunohistochemical, and molecular methods is often necessary for the precise diagnosis and optimal clinical management of Xp11.2 tRCCs.

As mentioned before, this disease has two distinct disease progression processes in different populations. Xp11.2 tRCC has an indolent course in children, while it commonly has an aggressive one in adult patients. In our study, three tumors affected children, and the oldest patient examined was 20 years old. This probably partially explained why most of our patients had good prognosis even with lymph node metastasis, except for the patient with the *VCP–TFE3* fusion. It should be noted that different fusion subtypes within the same translocation-associated neoplasm may not only influence biological features but also be associated with clinical outcomes. Previous studies showed that *PRCC–TFE3* presented at a lower stage and less metastatic frequency than did other tRCCs, such as *ASPL–TFE3* (33). The tendency for *PRCC–TFE3* tRCC to recur late warrants long-term follow-up. The same as in the literature, one of our patients with *ASPL–TFE3* fusion had local lymph node metastasis; the others with *ASPL–TFE3* and *PRCC–TFE3* tRCC had none. Our *VCP–TFE3* fusion patient came to us with multiple bone metastases at the first visit, which deceased 3 months later without any further treatment, probably indicating that *VCP–TFE3* has worse prognosis because of the fusion partner. Valosin-containing protein (VCP)/p97 (Cdc48) is a member of the ATP-binding protein family and is best known for its role in various important cellular events, such as protein homeostasis, but is also involved in regulating critical signaling pathways including cell cycle regulation. Additionally, clinical studies have identified a correlation between elevated VCP expression and the progression, prognosis, and metastatic potential of esophageal carcinoma (34), colorectal carcinoma (35), prostate cancer (36), non-small cell lung carcinoma (37), and pancreatic cancer. Furthermore, VCP was reported to be one of the few known recurrent amplicons at the DNA level associated with tumor metastasis (38), which is probably related to nuclear factor kappa B (NF-κB) signaling pathway (39). These findings probably propose the expression level of VCP as a useful marker for the progression of these cancers. In addition, a potential anti-apoptotic role of VCP was first described in 1991 by Shirogane et al. (40) and was further substantiated by the fact that increased levels of VCP strongly correlate with poor prognosis and metastasis of various human cancers. Our patient with the *VCP–TFE3* fusion had distant metastasis as an initial symptom and had a short survival period, which is probably related to the *VCP–TFE3* fusion. Although whether the fusion status is an independent predictor of outcomes is debated, these examples highlight the potential for the fusion subtype to impact patient care. Ellis et al. (33) mentioned a hypothesis that fusion types could highly possibly influence the tumor prognosis in Xp11.2 tRCCs with enough cases. Other researchers also noticed the same phenomenon in other malignant neoplasms such as Ewing sarcomas, primary mucoepidermoid carcinoma, and alveolar rhabdomyosarcomas. The type 1 *EWS–FLI1* fusion (fusion of exon 6 of *FLI1* with exon 7 of *EWS*) has better and favorable prognosis than other variant *EWS–FLI1* gene fusions (41–43). Furthermore, *CRTC3–MAML2* tends to have a smaller tumor size and better prognosis than does *CRTC1–MAML2*, although these data have no statistical significance because of the low number of *CRTC3–MAML2* cases (44, 45). Interestingly, the same is true for alveolar rhabdomyosarcomas. Cases with the *PAX3–FOXO1* gene fusion have worse outcome than those with the *PAX7–FOXO1* fusion (46). These different associations additionally support the notion that the fusion proteins encoded by these chromosomal translocations are probably central to the biology of these tumors.

Currently, the treatment for Xp11.2 tRCC is still unestablished, and no clinical studies with a large sample size are being conducted. Most cases followed the guidelines for conventional RCC. Surgical therapy plays a primary role and is currently focusing on organ preservation; however, this is based upon tumor localization and institutional experience. The therapy for metastatic Xp11.2 RCC is not different from that for conventional RCC. Therapies targeting vascular endothelial growth factor receptor (VEGF), mammalian target of rapamycin, and sorafenib may benefit patients with Xp11.2 tRCC (13). The prognosis is controversial since Xp11.2 tRCC has an indolent behavior in children; however, new reports on an aggressive clinical course in adults have been published as well (14). In multivariate analysis, only older age or an advanced stage at presentation predicted death. In our cohort, three patients were under 18 years old. The patient with *PRCC-TFE3* fusion was 20 years old. This *PRCC–TFE3* fusion patient and one of those with the *ASPL–TFE3* fusion (4 years old) were in the early stage and good condition, and one *ASPL-TFE3* RCC patient (9 years old) had lymph node metastasis. All three patients showed no recurrence after a 3-year follow-up. Our patient with the *VCP–TFE3* fusion (17 years old) had distant metastasis as an initial symptom and died of the disease after 3 months, probably indicating that the fusion type could influence prognosis.

Increased understanding of the clinical, pathological, molecular, and prognostic heterogeneity of Xp11.2 tRCC, since their official recognition in 2004, provides the opportunity to identify prognostic biomarkers and to understand the reasons for tumor aggression. The characteristics of these four cases showed some uniformity, but still have some differences. Immunohistochemistry results revealed tumor cells positive for *TFE3*, but have no consistency in other markers. Therefore, the uniform and definitive diagnostic standards of the tumors are uncertain. The prognosis of Xp11.2 RCC is still unclear because of the low appearance of series including a great number of patients and the short follow-up period. In this study, we discovered an unreported *VCP–TFE3* tRCC that had worse prognosis compared to other fusion Xp11.2 RCC. It is probable that the fusion type could influence therapy and prognosis. RNA sequencing probably needs to be done; even FISH detection has been conducted. Detection of the relationship between the gene changes and clinical parameters needs more cases and research. Hence, more cases and findings are required to elaborate the standards of all the tumor subtypes.

## Data Availability Statement

The original contributions presented in the study are included in the article. Further inquiries can be directed to the corresponding authors.

## Ethics Statement

The studies involving human participants were reviewed and approved by the Guangdong Provincial People’s Hospital Ethics Committee. Written informed consent to participate in this study was provided by the participants’ legal guardian/next of kin. Written informed consent was obtained from the individual(s) and minor(s)’ legal guardian/next of kin for the publication of any potentially identifiable images or data included in this article.

## Author Contributions

YG and XL were responsible for study conception and design. QZ, DL, and LL contributed to the acquisition of data (acquired and managed patients, provided facilities, etc.). YG wrote the manuscript. HW and ZL supervised the study. All authors read and approved the final manuscript. All authors contributed to the article and approved the submitted version.

## Conflict of Interest

The authors declare that the research was conducted in the absence of any commercial or financial relationships that could be construed as a potential conflict of interest.

## Publisher’s Note

All claims expressed in this article are solely those of the authors and do not necessarily represent those of their affiliated organizations, or those of the publisher, the editors and the reviewers. Any product that may be evaluated in this article, or claim that may be made by its manufacturer, is not guaranteed or endorsed by the publisher.
